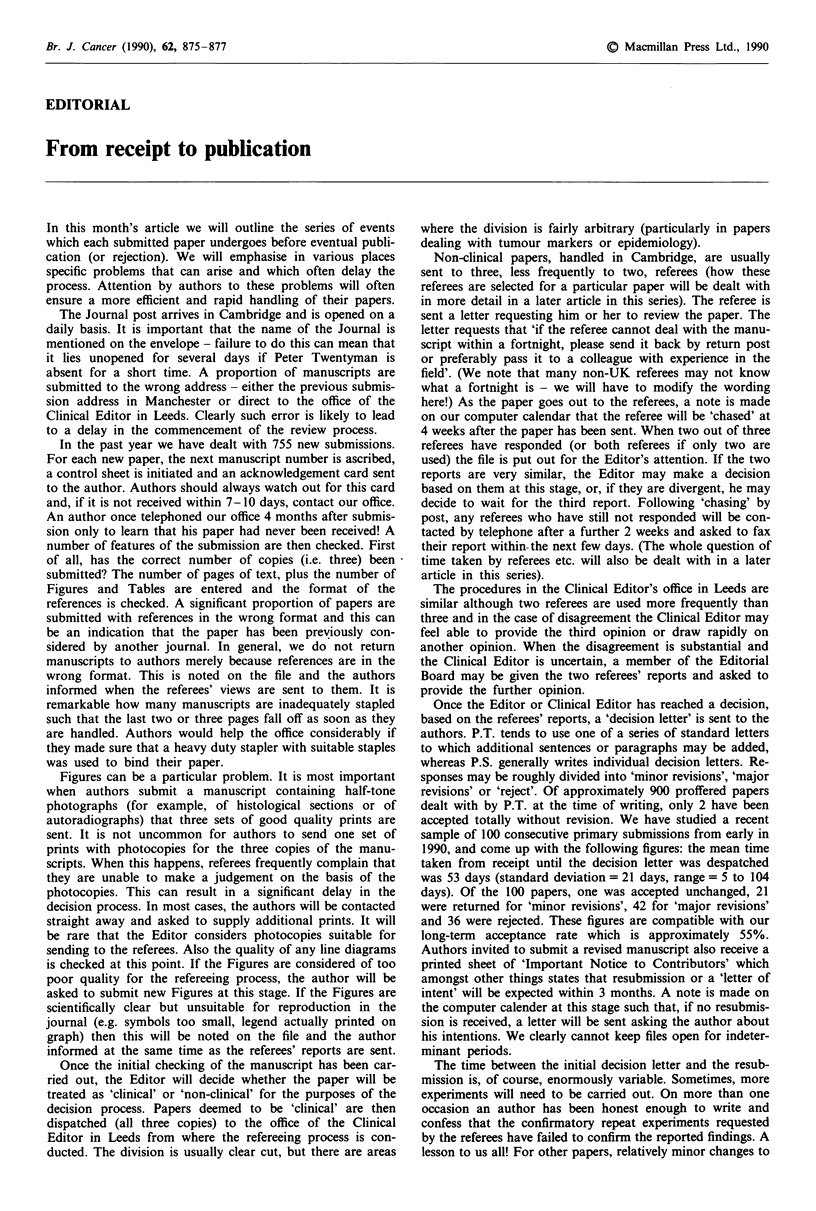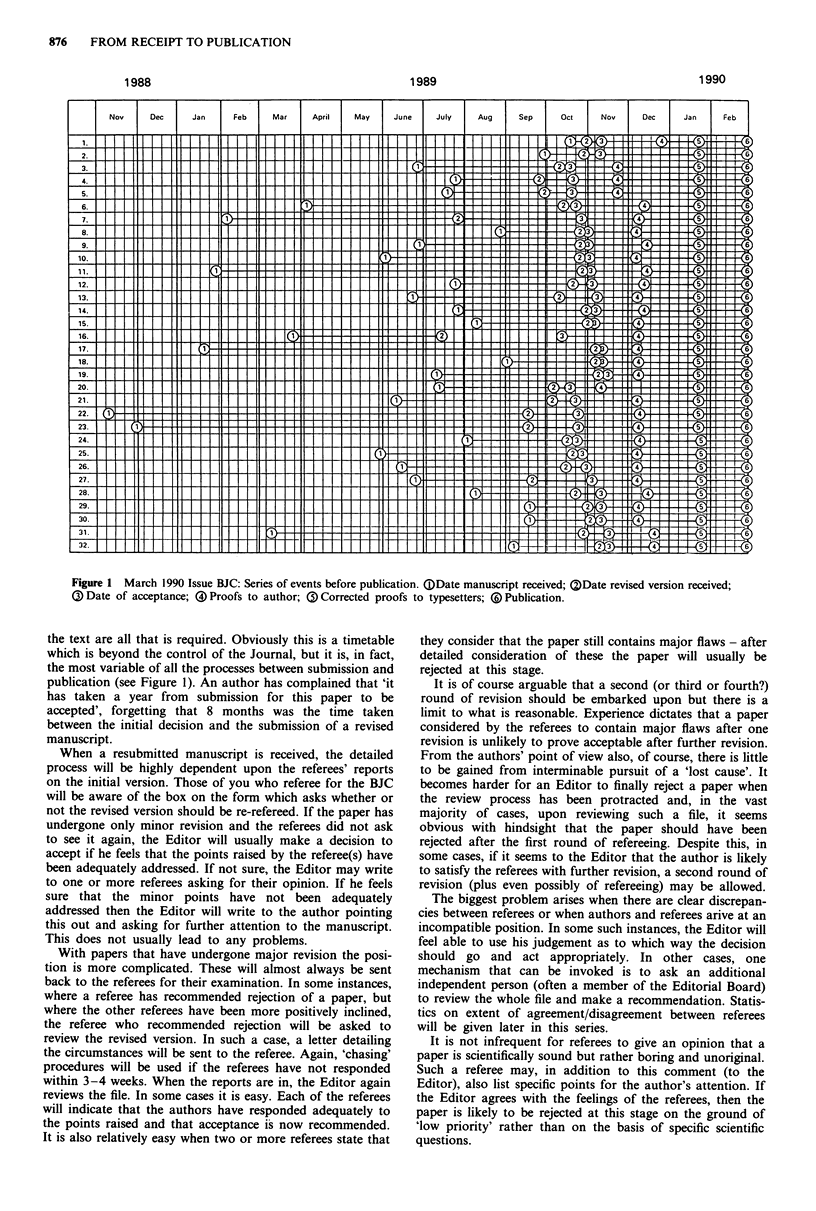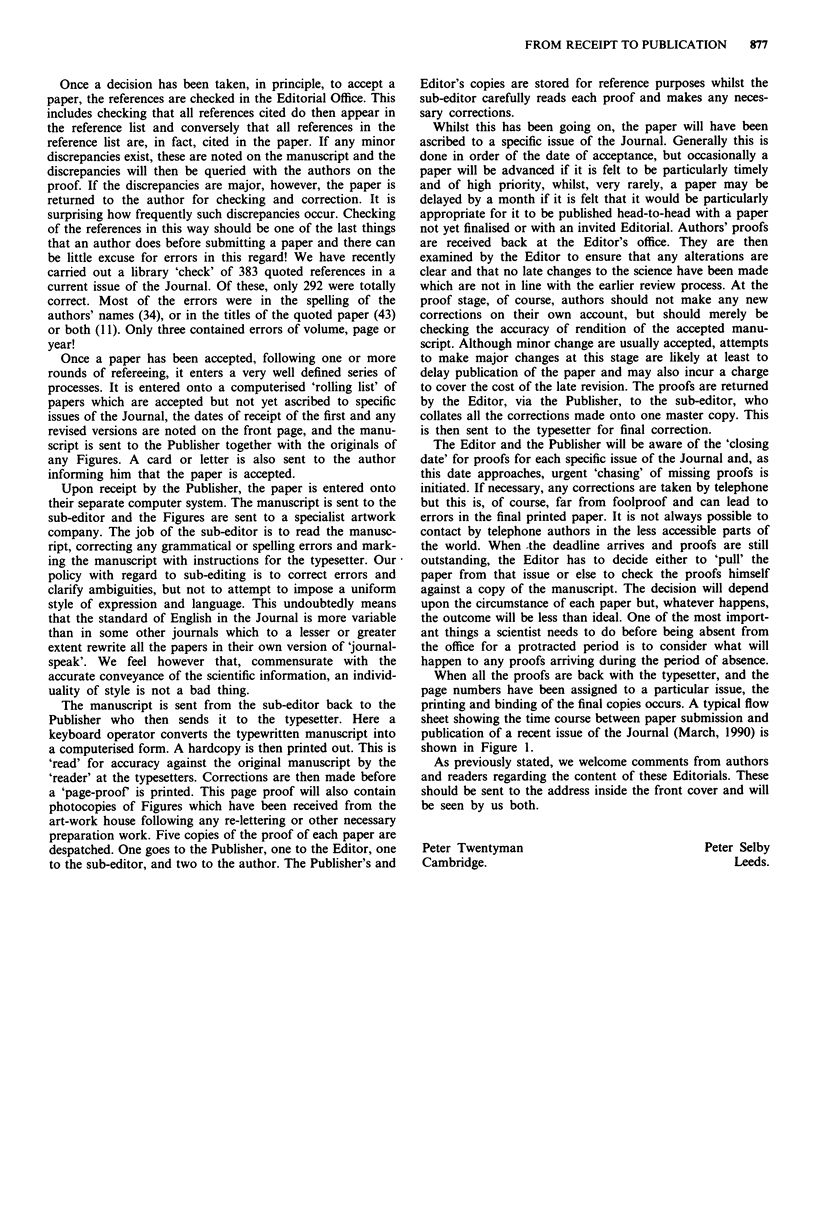# From receipt to publication.

**DOI:** 10.1038/bjc.1990.399

**Published:** 1990-12

**Authors:** P. Twentyman, P. Selby


*Br. J. Cancer* (1990), **62**, 875–877 © Macmillan Press Ltd., 1990

EDITORIAL

### From receipt to publication

In this month's article we will outline the series of events which each submitted paper undergoes before eventual publication (or rejection). We will emphasise in various places specific problems that can arise and which often delay the process. Attention by authors to these problems will often ensure a more efficient and rapid handling of their papers.

The Journal post arrives in Cambridge and is opened on a daily basis. It is important that the name of the Journal is mentioned on the envelope – failure to do this can mean that it lies unopened for several days if Peter Twentyman is absent for a short time. A proportion of manuscripts are submitted to the wrong address – either the previous submission address in Manchester or direct to the office of the Clinical Editor in Leeds. Clearly such error is likely to lead to a delay in the commencement of the review process.

In the past year we have dealt with 755 new submissions. For each new paper, the next manuscript number is ascribed, a control sheet is initiated and an acknowledgement card sent to the author. Authors should always watch out for this card and, if it is not received within 7–10 days, contact our office. An author once telephoned our office 4 months after submission only to learn that his paper had never been received! A number of features of the submission are then checked. First of all, has the correct number of copies (i.e. three) been submitted? The number of pages of text, plus the number of Figures and Tables are entered and the format of the references is checked. A significant proportion of papers are submitted with references in the wrong format and this can be an indication that the paper has been previously considered by another journal. In general, we do not return manuscripts to authors merely because references are in the wrong format. This is noted on the file and the authors informed when the referees' views are sent to them. It is remarkable how many manuscripts are inadequately stapled such that the last two or three pages fall off as soon as they are handled. Authors would help the office considerably if they made sure that a heavy duty stapler with suitable staples was used to bind their paper.

Figures can be a particular problem. It is most important when authors submit a manuscript containing half-tone photographs (for example, of histological sections or of autoradiographs) that three sets of good quality prints are sent. It is not uncommon for authors to send one set of prints with photocopies for the three copies of the manuscripts. When this happens, referees frequently complain that they are unable to make a judgement on the basis of the photocopies. This can result in a significant delay in the decision process. In most cases, the authors will be contacted straight away and asked to supply additional prints. It will be rare that the Editor considers photocopies suitable for sending to the referees. Also the quality of any line diagrams is checked at this point. If the Figures are considered of too poor quality for the refereeing process, the author will be asked to submit new Figures at this stage. If the Figures are scientifically clear but unsuitable for reproduction in the journal (e.g. symbols too small, legend actually printed on graph) then this will be noted on the file and the author informed at the same time as the referees' reports are sent.

Once the initial checking of the manuscript has been carried out, the Editor will decide whether the paper will be treated as 'clinical' or 'non-clinical' for the purposes of the decision process. Papers deemed to be 'clinical' are then dispatched (all three copies) to the office of the Clinical Editor in Leeds from where the refereeing process is conducted. The division is usually clear cut, but there are areas where the division is fairly arbitrary (particularly in papers dealing with tumour markers or epidemiology).

Non-clinical papers, handled in Cambridge, are usually sent to three, less frequently to two, referees (how these referees are selected for a particular paper will be dealt with in more detail in a later article in this series). The referee is sent a letter requesting him or her to review the paper. The letter requests that 'if the referee cannot deal with the manuscript within a fortnight, please send it back by return post or preferably pass it to a colleague with experience in the field'. (We note that many non-UK referees may not know what a fortnight is – we will have to modify the wording here!) As the paper goes out to the referees, a note is made on our computer calendar that the referee will be 'chased' at 4 weeks after the paper has been sent. When two out of three referees have responded (or both referees if only two are used) the file is put out for the Editor's attention. If the two reports are very similar, the Editor may make a decision based on them at this stage, or, if they are divergent, he may decide to wait for the third report. Following 'chasing' by post, any referees who have still not responded will be contacted by telephone after a further 2 weeks and asked to fax their report within the next few days. (The whole question of time taken by referees etc. will also be dealt with in a later article in this series).

The procedures in the Clinical Editor's office in Leeds are similar although two referees are used more frequently than three and in the case of disagreement the Clinical Editor may feel able to provide the third opinion or draw rapidly on another opinion. When the disagreement is substantial and the Clinical Editor is uncertain, a member of the Editorial Board may be given the two referees' reports and asked to provide the further opinion.

Once the Editor or Clinical Editor has reached a decision, based on the referees' reports, a 'decision letter' is sent to the authors. P.T. tends to use one of a series of standard letters to which additional sentences or paragraphs may be added, whereas P.S. generally writes individual decision letters. Responses may be roughly divided into 'minor revisions', 'major revisions' or 'reject'. Of approximately 900 proffered papers dealt with by P.T. at the time of writing, only 2 have been accepted totally without revision. We have studied a recent sample of 100 consecutive primary submissions from early in 1990, and come up with the following figures: the mean time taken from receipt until the decision letter was despatched was 53 days (standard deviation = 21 days, range = 5 to 104 days). Of the 100 papers, one was accepted unchanged, 21 were returned for 'minor revisions', 42 for 'major revisions' and 36 were rejected. These figures are compatible with our long-term acceptance rate which is approximately 55%. Authors invited to submit a revised manuscript also receive a printed sheet of 'Important Notice to Contributors' which amongst other things states that resubmission or a 'letter of intent' will be expected within 3 months. A note is made on the computer calender at this stage such that, if no resubmission is received, a letter will be sent asking the author about his intentions. We clearly cannot keep files open for indeterminant periods.

The time between the initial decision letter and the resubmission is, of course, enormously variable. Sometimes, more experiments will need to be carried out. On more than one occasion an author has been honest enough to write and confess that the confirmatory repeat experiments requested by the referees have failed to confirm the reported findings. A lesson to us all! For other papers, relatively minor changes to

**Figure 1** March 1990 Issue BJC: Series of events before publication. ① Date manuscript received; ② Date revised version received; ③ Date of acceptance; ④ Proofs to author; ⑤ Corrected proofs to typesetters; ⑥ Publication.

the text are all that is required. Obviously this is a timetable which is beyond the control of the Journal, but it is, in fact, the most variable of all the processes between submission and publication (see Figure 1). An author has complained that 'it has taken a year from submission for this paper to be accepted', forgetting that 8 months was the time taken between the initial decision and the submission of a revised manuscript.

When a resubmitted manuscript is received, the detailed process will be highly dependent upon the referees' reports on the initial version. Those of you who referee for the BJC will be aware of the box on the form which asks whether or not the revised version should be re-refereed. If the paper has undergone only minor revision and the referees did not ask to see it again, the Editor will usually make a decision to accept if he feels that the points raised by the referee(s) have been adequately addressed. If not sure, the Editor may write to one or more referees asking for their opinion. If he feels sure that the minor points have not been adequately addressed then the Editor will write to the author pointing this out and asking for further attention to the manuscript. This does not usually lead to any problems.

With papers that have undergone major revision the position is more complicated. These will almost always be sent back to the referees for their examination. In some instances, where a referee has recommended rejection of a paper, but where the other referees have been more positively inclined, the referee who recommended rejection will be asked to review the revised version. In such a case, a letter detailing the circumstances will be sent to the referee. Again, 'chasing' procedures will be used if the referees have not responded within 3–4 weeks. When the reports are in, the Editor again reviews the file. In some cases it is easy. Each of the referees will indicate that the authors have responded adequately to the points raised and that acceptance is now recommended. It is also relatively easy when two or more referees state that they consider that the paper still contains major flaws – after detailed consideration of these the paper will usually be rejected at this stage.

It is of course arguable that a second (or third or fourth?) round of revision should be embarked upon but there is a limit to what is reasonable. Experience dictates that a paper considered by the referees to contain major flaws after one revision is unlikely to prove acceptable after further revision. From the authors' point of view also, of course, there is little to be gained from interminable pursuit of a 'lost cause'. It becomes harder for an Editor to finally reject a paper when the review process has been protracted and, in the vast majority of cases, upon reviewing such a file, it seems obvious with hindsight that the paper should have been rejected after the first round of refereeing. Despite this, in some cases, if it seems to the Editor that the author is likely to satisfy the referees with further revision, a second round of revision (plus even possibly of refereeing) may be allowed.

The biggest problem arises when there are clear discrepancies between referees or when authors and referees arive at an incompatible position. In some such instances, the Editor will feel able to use his judgement as to which way the decision should go and act appropriately. In other cases, one mechanism that can be invoked is to ask an additional independent person (often a member of the Editorial Board) to review the whole file and make a recommendation. Statistics on extent of agreement/disagreement between referees will be given later in this series.

It is not infrequent for referees to give an opinion that a paper is scientifically sound but rather boring and unoriginal. Such a referee may, in addition to this comment (to the Editor), also list specific points for the author's attention. If the Editor agrees with the feelings of the referees, then the paper is likely to be rejected at this stage on the ground of 'low priority' rather than on the basis of specific scientific questions.

Once a decision has been taken, in principle, to accept a paper, the references are checked in the Editorial Office. This includes checking that all references cited do then appear in the reference list and conversely that all references in the reference list are, in fact, cited in the paper. If any minor discrepancies exist, these are noted on the manuscript and the discrepancies will then be queried with the authors on the proof. If the discrepancies are major, however, the paper is returned to the author for checking and correction. It is surprising how frequently such discrepancies occur. Checking of the references in this way should be one of the last things that an author does before submitting a paper and there can be little excuse for errors in this regard! We have recently carried out a library 'check' of 383 quoted references in a current issue of the Journal. Of these, only 292 were totally correct. Most of the errors were in the spelling of the authors' names (34), or in the titles of the quoted paper (43) or both (11). Only three contained errors of volume, page or year!

Once a paper has been accepted, following one or more rounds of refereeing, it enters a very well defined series of processes. It is entered onto a computerised 'rolling list' of papers which are accepted but not yet ascribed to specific issues of the Journal, the dates of receipt of the first and any revised versions are noted on the front page, and the manuscript is sent to the Publisher together with the originals of any Figures. A card or letter is also sent to the author informing him that the paper is accepted.

Upon receipt by the Publisher, the paper is entered onto their separate computer system. The manuscript is sent to the sub-editor and the Figures are sent to a specialist artwork company. The job of the sub-editor is to read the manuscript, correcting any grammatical or spelling errors and marking the manuscript with instructions for the typesetter. Our policy with regard to sub-editing is to correct errors and clarify ambiguities, but not to attempt to impose a uniform style of expression and language. This undoubtedly means that the standard of English in the Journal is more variable than in some other journals which to a lesser or greater extent rewrite all the papers in their own version of 'journal-speak'. We feel however that, commensurate with the accurate conveyance of the scientific information, an individuality of style is not a bad thing.

The manuscript is sent from the sub-editor back to the Publisher who then sends it to the typesetter. Here a keyboard operator converts the typewritten manuscript into a computerised form. A hardcopy is then printed out. This is 'read' for accuracy against the original manuscript by the 'reader' at the typesetters. Corrections are then made before a 'page-proof' is printed. This page proof will also contain photocopies of Figures which have been received from the art-work house following any re-lettering or other necessary preparation work. Five copies of the proof of each paper are despatched. One goes to the Publisher, one to the Editor, one to the sub-editor, and two to the author. The Publisher's and Editor's copies are stored for reference purposes whilst the sub-editor carefully reads each proof and makes any necessary corrections.

Whilst this has been going on, the paper will have been ascribed to a specific issue of the Journal. Generally this is done in order of the date of acceptance, but occasionally a paper will be advanced if it is felt to be particularly timely and of high priority, whilst, very rarely, a paper may be delayed by a month if it is felt that it would be particularly appropriate for it to be published head-to-head with a paper not yet finalised or with an invited Editorial. Authors' proofs are received back at the Editor's office. They are then examined by the Editor to ensure that any alterations are clear and that no late changes to the science have been made which are not in line with the earlier review process. At the proof stage, of course, authors should not make any new corrections on their own account, but should merely be checking the accuracy of rendition of the accepted manuscript. Although minor change are usually accepted, attempts to make major changes at this stage are likely at least to delay publication of the paper and may also incur a charge to cover the cost of the late revision. The proofs are returned by the Editor, via the Publisher, to the sub-editor, who collates all the corrections made onto one master copy. This is then sent to the typesetter for final correction.

The Editor and the Publisher will be aware of the 'closing date' for proofs for each specific issue of the Journal and, as this date approaches, urgent 'chasing' of missing proofs is initiated. If necessary, any corrections are taken by telephone but this is, of course, far from foolproof and can lead to errors in the final printed paper. It is not always possible to contact by telephone authors in the less accessible parts of the world. When the deadline arrives and proofs are still outstanding, the Editor has to decide either to 'pull' the paper from that issue or else to check the proofs himself against a copy of the manuscript. The decision will depend upon the circumstance of each paper but, whatever happens, the outcome will be less than ideal. One of the most important things a scientist needs to do before being absent from the office for a protracted period is to consider what will happen to any proofs arriving during the period of absence.

When all the proofs are back with the typesetter, and the page numbers have been assigned to a particular issue, the printing and binding of the final copies occurs. A typical flow sheet showing the time course between paper submission and publication of a recent issue of the Journal (March, 1990) is shown in Figure 1.

As previously stated, we welcome comments from authors and readers regarding the content of these Editorials. These should be sent to the address inside the front cover and will be seen by us both.

Peter Twentyman

Cambridge.

Peter Selby